# Poly[bis­(μ_2_-benzyl­oxyacetato-κ^3^ *O*,*O*′:*O*′′)cadmium(II)]

**DOI:** 10.1107/S1600536808010799

**Published:** 2008-04-23

**Authors:** Ji-Wei Liu, Seik Weng Ng

**Affiliations:** a College of Chemistry and Chemical Technology, Daqing Petroleum Institute, Daqing 163318, People’s Republic of China; b Department of Chemistry, University of Malaya, 50603 Kuala Lumpur, Malaysia

## Abstract

The title cadmium derivative of benzyl­oxyacetic acid, [Cd(C_9_H_9_O_3_)_2_]_*n*_, exists as a *μ*_2_-carboxyl­ate-bridged layer network. Two benzyl­oxyacetate units each chelate the metal through a carboxylate as well as through the ether O atoms; the metal is also coordinated by the double-bond carbonyl O atom of two adjacent benzyl­oxyacetate units in an octa­hedral geometry. The metal atom lies on a special position of 2 site symmetry. The phenyl group is disordered equally over two positions.

## Related literature

There are no crystallographic examples of metal benzyl­oxyacetates although there are many examples of metal aryl­oxyacetates. For mononuclear diaquadi(phenoxy­acetato)cadmium, see: Mak *et al.* (1985 ▶).
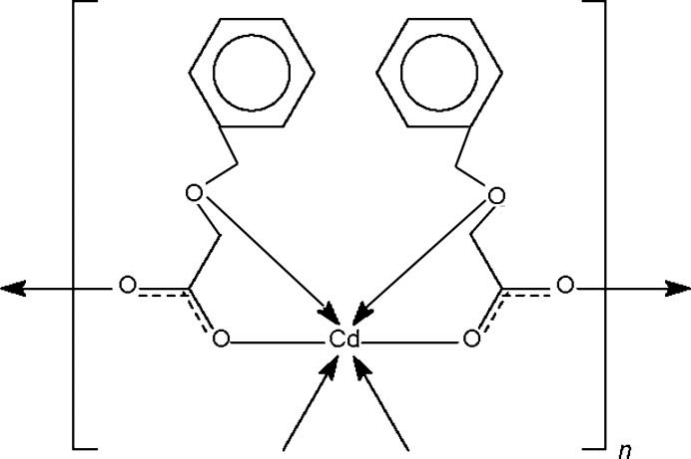


## Experimental

### 

#### Crystal data

[Cd(C_9_H_9_O_3_)_2_]
*M* *_r_* = 442.72
Orthorhombic, 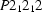

*a* = 6.7430 (2) Å
*b* = 8.9449 (2) Å
*c* = 15.4736 (4) Å
*V* = 933.30 (4) Å^3^
*Z* = 2
Mo *K*α radiation
μ = 1.20 mm^−1^
*T* = 295 (2) K
0.33 × 0.13 × 0.04 mm

#### Data collection

Bruker APEXII diffractometer
Absorption correction: multi-scan (*SADABS*; Sheldrick, 1996 ▶) *T*_min_ = 0.693, *T*_max_ = 0.954
5780 measured reflections
1639 independent reflections
1483 reflections with *I* > 2σ(*I*)
*R*_int_ = 0.039

#### Refinement

*R*[*F* ^2^ > 2σ(*F* ^2^)] = 0.044
*wR*(*F* ^2^) = 0.124
*S* = 1.09
1639 reflections
108 parameters
37 restraints
H-atom parameters constrained
Δρ_max_ = 2.22 e Å^−3^
Δρ_min_ = −0.88 e Å^−3^
Absolute structure: Flack (1983 ▶), 501 Friedel pairs
Flack parameter: 0.03 (8)

### 

Data collection: *APEX2* (Bruker, 2006 ▶); cell refinement: *SAINT* (Bruker, 2006 ▶); data reduction: *SAINT*; program(s) used to solve structure: *SHELXS97* (Sheldrick, 2008 ▶); program(s) used to refine structure: *SHELXL97* (Sheldrick, 2008 ▶); molecular graphics: *X-SEED* (Barbour, 2001 ▶); *OLEX* (Dolomanov *et al.*, 2003 ▶); software used to prepare material for publication: *SHELXL97*.

## Supplementary Material

Crystal structure: contains datablocks global, I. DOI: 10.1107/S1600536808010799/sg2235sup1.cif

Structure factors: contains datablocks I. DOI: 10.1107/S1600536808010799/sg2235Isup2.hkl

Additional supplementary materials:  crystallographic information; 3D view; checkCIF report

## Figures and Tables

**Table:** 

Cd1—O1	2.268 (4)
Cd1—O1^i^	2.268 (4)
Cd1—O2^ii^	2.226 (5)
Cd1—O2^iii^	2.226 (5)
Cd1—O3	2.379 (4)
Cd1—O3^i^	2.379 (4)

**Table:** 

O1—Cd1—O1^i^	161.2 (3)
O1—Cd1—O2^ii^	105.9 (2)
O1—Cd1—O2^iii^	87.2 (2)
O1—Cd1—O3	69.6 (2)
O1—Cd1—O3^i^	95.5 (2)
O1^i^—Cd1—O2^ii^	87.2 (2)
O1^i^—Cd1—O2^iii^	105.9 (2)
O1^i^—Cd1—O3^i^	69.6 (2)
O1^i^—Cd1—O3	95.5 (2)
O2^ii^—Cd1—O2^iii^	93.3 (3)
O2^ii^—Cd1—O3	98.3 (2)
O2^ii^—Cd1—O3^i^	156.2 (2)
O2^iii^—Cd1—O3	156.2 (2)
O2^iii^—Cd1—O3^i^	98.3 (2)
O3—Cd1—O3^i^	79.1 (2)

Symmetry codes: (i) 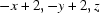; (ii) 
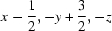
; (iii) 
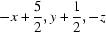
.

**Fig. 1.**
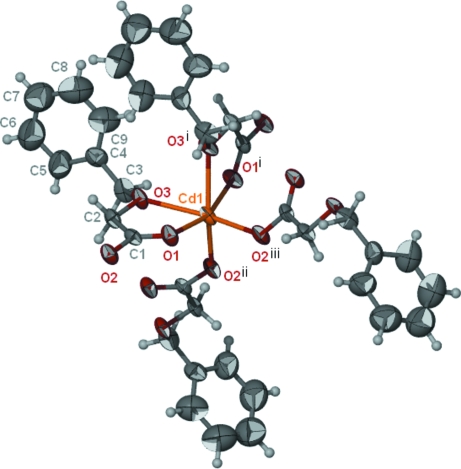
Thermal displacement ellipsoid plot (Barbour, 2001) illustrating the coordination geometry of Cd in [Cd(C9H9O3)2]n. Displacement ellipsoids are drawn at the 50% probability level and H atoms as spheres of arbitrary radii.

**Fig. 2.**
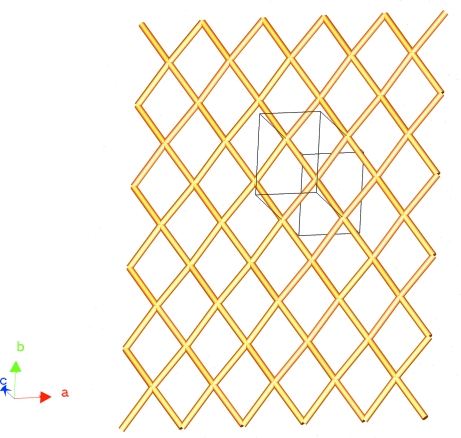
OLEX (Dolomanov et al., 2003) representation of the layer structure.

## supplementary crystallographic information

### Comment

The crystal structures of a large number of metal derivatives of aryloxyacetic acids have been reported; in some structures, the ether oxygen also engages in bonding so that the carboxylate unit functions both as a chelate as well as a bridge. The cadmium derivative of phenyoxyacetic acid exists as a diaqua, carboxylate-chelated compound. The carboxyl –CO_2_ portion engages in chelation instead (Mak *et al.*, 1985). The title cadmium analog has a benzyl group in place of the phenyl group, which is probably less crowded; this feature permits the ether linkage to bind to the metal atom. The compound (Scheme I) is an anhydrous compound; the carboxylate group chelates to the metal atom. It also bridges adjacent metal atoms (Fig. 1); the bridges lead to the formation of a layer motif (Fig. 2).

### Experimental

Cadmium dinitrate tetrahydrate (0.31 g, 1 mmol) and 2,2'-bipyridine (0.16 g, 1 mmol) were added to a hot aqueous solution of benzyloxyacetic acid (0.17 g, 1 mmol). The pH of the solution was adjusted to 6 with 0.1 *M* sodium hydroxide. The solution was allowed to evaporate at room temperature. Colorless single crystals are separated from the filtered solution after several days.

### Refinement

Hydrogen atoms were treated as riding, with C–H = 0.93 to 0.97 Å and were included in the refinement with *U*(H) set to 1.2 times *U*_eq_(C). The phenyl ring is disordered over two sites; the occupancy could not be refined, and each component was arbitrarily assigned 0.5 occupancy. The ring was refined as a rigid hexagon; the temperature factors of the primed atoms were constrained to those of the unprimed ones. The anisotropic temperature factors of the ring were retrained to be nearly isotropic. The C3–C4 and C3–C4' distances were restrained to within 0.01 Å of each other.

The final difference Fourier map had a large peak at about 1 Å from Cd1.

### Crystal data

**Table:**

[Cd(C_9_H_9_O_3_)_2_]	*F*_000_ = 444
*M**_r_* = 442.72	*D*_x_ = 1.575 Mg m^−^^3^
Orthorhombic, *P*2_1_2_1_2	Mo *K*α radiation λ = 0.71073 Å
Hall symbol: P 2 2ab	Cell parameters from 2270 reflections
*a* = 6.7430 (2) Å	θ = 2.6–23.6º
*b* = 8.9449 (2) Å	µ = 1.20 mm^−^^1^
*c* = 15.4736 (4) Å	*T* = 295 (2) K
*V* = 933.30 (4) Å^3^	Block, colorless
*Z* = 2	0.33 × 0.13 × 0.04 mm

### Data collection

**Table:**

Bruker APEXII diffractometer	1639 independent reflections
Radiation source: fine-focus sealed tube	1483 reflections with *I* > 2σ(*I*)
Monochromator: graphite	*R*_int_ = 0.039
*T* = 295(2) K	θ_max_ = 25.0º
φ and ω scans	θ_min_ = 1.3º
Absorption correction: multi-scan(SADABS; Sheldrick, 1996)	*h* = −7→7
*T*_min_ = 0.693, *T*_max_ = 0.954	*k* = −10→9
5780 measured reflections	*l* = −18→17

### Refinement

**Table:**

Refinement on *F*^2^	Hydrogen site location: inferred from neighbouring sites
Least-squares matrix: full	H-atom parameters constrained
*R*[*F*^2^ > 2σ(*F*^2^)] = 0.044	*w* = 1/[σ^2^(*F*_o_^2^) + (0.0833*P*)^2^] where *P* = (*F*_o_^2^ + 2*F*_c_^2^)/3
*wR*(*F*^2^) = 0.125	(Δ/σ)_max_ = 0.001
*S* = 1.09	Δρ_max_ = 2.22 e Å^−^^3^
1639 reflections	Δρ_min_ = −0.88 e Å^−^^3^
108 parameters	Extinction correction: none
37 restraints	Absolute structure: Flack (1983), 501 Friedel pairs
Primary atom site location: structure-invariant direct methods	Flack parameter: 0.03 (8)
Secondary atom site location: difference Fourier map	

### Fractional atomic coordinates and isotropic or equivalent isotropic displacement parameters (Å^2^)

**Table:**

	*x*	*y*	*z*	*U*_iso_*/*U*_eq_	Occ. (<1)
Cd1	1.0000	1.0000	0.01184 (4)	0.0405 (3)	
O1	1.2315 (7)	0.8208 (5)	0.0358 (3)	0.0481 (12)	
O2	1.2948 (7)	0.5938 (5)	0.0869 (3)	0.0495 (12)	
O3	0.9048 (6)	0.8466 (5)	0.1304 (3)	0.0443 (11)	
C1	1.1913 (10)	0.7127 (7)	0.0819 (4)	0.0385 (15)	
C2	1.0100 (14)	0.7103 (6)	0.1367 (4)	0.0427 (13)	
H2A	0.9248	0.6287	0.1186	0.051*	
H2B	1.0472	0.6931	0.1964	0.051*	
C3	0.7017 (11)	0.8363 (9)	0.1615 (5)	0.059 (2)	
H3A	0.6423	0.7463	0.1381	0.070*	
H3B	0.6276	0.9209	0.1392	0.070*	
C4	0.680 (3)	0.834 (2)	0.2552 (5)	0.060 (3)	0.50
C5	0.567 (2)	0.7180 (17)	0.2895 (9)	0.100 (5)	0.50
H5	0.5061	0.6494	0.2529	0.119*	0.50
C6	0.545 (3)	0.705 (2)	0.3785 (10)	0.124 (6)	0.50
H6	0.4689	0.6276	0.4014	0.149*	0.50
C7	0.636 (3)	0.807 (2)	0.4332 (5)	0.127 (8)	0.50
H7	0.6206	0.7986	0.4927	0.152*	0.50
C8	0.749 (4)	0.923 (2)	0.3989 (9)	0.135 (5)	0.50
H8	0.8096	0.9915	0.4355	0.162*	0.50
C9	0.771 (4)	0.936 (2)	0.3099 (10)	0.108 (5)	0.50
H9	0.8468	1.0133	0.2870	0.130*	0.50
C4'	0.704 (3)	0.8030 (19)	0.2550 (5)	0.060 (3)	0.50
C5'	0.682 (3)	0.6579 (16)	0.2856 (9)	0.100 (5)	0.50
H5'	0.6602	0.5798	0.2471	0.119*	0.50
C6'	0.691 (3)	0.6296 (17)	0.3739 (10)	0.124 (6)	0.50
H6'	0.6755	0.5325	0.3943	0.149*	0.50
C7'	0.723 (3)	0.746 (2)	0.4315 (6)	0.127 (8)	0.50
H7'	0.7288	0.7273	0.4905	0.152*	0.50
C8'	0.745 (4)	0.891 (2)	0.4009 (9)	0.135 (5)	0.50
H8'	0.7667	0.9695	0.4395	0.162*	0.50
C9'	0.736 (4)	0.9198 (16)	0.3127 (10)	0.108 (5)	0.50
H9'	0.7514	1.0168	0.2922	0.130*	0.50

### Atomic displacement parameters (Å^2^)

**Table:**

	*U*^11^	*U*^22^	*U*^33^	*U*^12^	*U*^13^	*U*^23^
Cd1	0.0336 (4)	0.0237 (3)	0.0641 (4)	0.0012 (3)	0.000	0.000
O1	0.041 (3)	0.033 (3)	0.070 (3)	0.010 (2)	0.012 (2)	0.006 (3)
O2	0.048 (3)	0.030 (2)	0.070 (3)	0.010 (2)	0.006 (2)	0.003 (2)
O3	0.037 (2)	0.028 (2)	0.068 (3)	0.0084 (19)	0.012 (2)	0.008 (2)
C1	0.036 (4)	0.027 (3)	0.053 (4)	0.006 (3)	−0.003 (3)	−0.005 (3)
C2	0.037 (3)	0.028 (3)	0.063 (3)	0.007 (4)	−0.006 (6)	0.004 (2)
C3	0.033 (4)	0.056 (4)	0.087 (6)	0.016 (4)	0.015 (4)	0.008 (4)
C4	0.054 (6)	0.054 (6)	0.072 (5)	0.009 (6)	0.014 (4)	−0.012 (4)
C5	0.109 (10)	0.098 (9)	0.092 (7)	0.006 (7)	0.031 (7)	0.016 (7)
C6	0.126 (11)	0.126 (9)	0.120 (8)	−0.006 (8)	0.019 (8)	0.020 (8)
C7	0.132 (11)	0.134 (11)	0.114 (9)	0.012 (9)	0.018 (8)	−0.004 (8)
C8	0.134 (9)	0.152 (9)	0.119 (8)	−0.005 (8)	−0.001 (7)	−0.031 (7)
C9	0.091 (9)	0.122 (8)	0.112 (7)	−0.008 (7)	0.007 (6)	−0.025 (6)
C4'	0.054 (6)	0.054 (6)	0.072 (5)	0.009 (6)	0.014 (4)	−0.012 (4)
C5'	0.109 (10)	0.098 (9)	0.092 (7)	0.006 (7)	0.031 (7)	0.016 (7)
C6'	0.126 (11)	0.126 (9)	0.120 (8)	−0.006 (8)	0.019 (8)	0.020 (8)
C7'	0.132 (11)	0.134 (11)	0.114 (9)	0.012 (9)	0.018 (8)	−0.004 (8)
C8'	0.134 (9)	0.152 (9)	0.119 (8)	−0.005 (8)	−0.001 (7)	−0.031 (7)
C9'	0.091 (9)	0.122 (8)	0.112 (7)	−0.008 (7)	0.007 (6)	−0.025 (6)

### Geometric parameters (Å, °)

**Table:**

Cd1—O1	2.268 (4)	C5—C6	1.3900
Cd1—O1^i^	2.268 (4)	C5—H5	0.9300
Cd1—O2^ii^	2.226 (5)	C6—C7	1.3900
Cd1—O2^iii^	2.226 (5)	C6—H6	0.9300
Cd1—O3	2.379 (4)	C7—C8	1.3900
Cd1—O3^i^	2.379 (4)	C7—H7	0.9300
O1—C1	1.232 (8)	C8—C9	1.3900
O2—C1	1.274 (7)	C8—H8	0.9300
O2—Cd1^iv^	2.226 (5)	C9—H9	0.9300
O3—C2	1.414 (7)	C4'—C5'	1.3900
O3—C3	1.455 (8)	C4'—C9'	1.3900
C1—C2	1.488 (11)	C5'—C6'	1.3900
C2—H2A	0.9700	C5'—H5'	0.9300
C2—H2B	0.9700	C6'—C7'	1.3900
C3—C4	1.457 (11)	C6'—H6'	0.9300
C3—C4'	1.477 (11)	C7'—C8'	1.3900
C3—H3A	0.9700	C7'—H7'	0.9300
C3—H3B	0.9700	C8'—C9'	1.3900
C4—C5	1.3900	C8'—H8'	0.9300
C4—C9	1.3900	C9'—H9'	0.9300
			
O1—Cd1—O1^i^	161.2 (3)	H3A—C3—H3B	107.5
O1—Cd1—O2^ii^	105.9 (2)	C5—C4—C9	120.0
O1—Cd1—O2^iii^	87.2 (2)	C5—C4—C3	116.6 (12)
O1—Cd1—O3	69.6 (2)	C9—C4—C3	123.4 (12)
O1—Cd1—O3^i^	95.5 (2)	C6—C5—C4	120.0
O1^i^—Cd1—O2^ii^	87.2 (2)	C6—C5—H5	120.0
O1^i^—Cd1—O2^iii^	105.9 (2)	C4—C5—H5	120.0
O1^i^—Cd1—O3^i^	69.6 (2)	C5—C6—C7	120.0
O1^i^—Cd1—O3	95.5 (2)	C5—C6—H6	120.0
O2^ii^—Cd1—O2^iii^	93.3 (3)	C7—C6—H6	120.0
O2^ii^—Cd1—O3	98.3 (2)	C6—C7—C8	120.0
O2^ii^—Cd1—O3^i^	156.2 (2)	C6—C7—H7	120.0
O2^iii^—Cd1—O3	156.2 (2)	C8—C7—H7	120.0
O2^iii^—Cd1—O3^i^	98.3 (2)	C9—C8—C7	120.0
O3—Cd1—O3^i^	79.1 (2)	C9—C8—H8	120.0
C1—O1—Cd1	119.9 (4)	C7—C8—H8	120.0
C1—O2—Cd1^iv^	127.8 (5)	C8—C9—C4	120.0
C2—O3—C3	113.3 (6)	C8—C9—H9	120.0
C2—O3—Cd1	114.5 (4)	C4—C9—H9	120.0
C3—O3—Cd1	123.1 (4)	C5'—C4'—C9'	120.0
O1—C1—O2	124.7 (6)	C5'—C4'—C3	121.4 (12)
O1—C1—C2	121.5 (5)	C9'—C4'—C3	118.6 (12)
O2—C1—C2	113.8 (6)	C6'—C5'—C4'	120.0
O3—C2—C1	111.1 (5)	C6'—C5'—H5'	120.0
O3—C2—H2A	109.4	C4'—C5'—H5'	120.0
C1—C2—H2A	109.4	C5'—C6'—C7'	120.0
O3—C2—H2B	109.4	C5'—C6'—H6'	120.0
C1—C2—H2B	109.4	C7'—C6'—H6'	120.0
H2A—C2—H2B	108.0	C6'—C7'—C8'	120.0
O3—C3—C4	115.1 (10)	C6'—C7'—H7'	120.0
O3—C3—C4'	109.0 (11)	C8'—C7'—H7'	120.0
O3—C3—H3A	108.5	C9'—C8'—C7'	120.0
C4—C3—H3A	108.5	C9'—C8'—H8'	120.0
C4'—C3—H3A	101.8	C7'—C8'—H8'	120.0
O3—C3—H3B	108.5	C8'—C9'—C4'	120.0
C4—C3—H3B	108.5	C8'—C9'—H9'	120.0
C4'—C3—H3B	120.9	C4'—C9'—H9'	120.0
			
O2^ii^—Cd1—O1—C1	−78.1 (6)	Cd1—O3—C2—C1	15.2 (7)
O2^iii^—Cd1—O1—C1	−170.7 (5)	O1—C1—C2—O3	−2.7 (10)
O1^i^—Cd1—O1—C1	54.5 (5)	O2—C1—C2—O3	178.2 (6)
O3^i^—Cd1—O1—C1	91.2 (6)	C2—O3—C3—C4	76.0 (10)
O3—Cd1—O1—C1	15.0 (5)	Cd1—O3—C3—C4	−139.0 (9)
O2^ii^—Cd1—O3—C2	88.3 (5)	C2—O3—C3—C4'	64.4 (10)
O2^iii^—Cd1—O3—C2	−29.9 (7)	Cd1—O3—C3—C4'	−150.7 (8)
O1^i^—Cd1—O3—C2	176.3 (5)	O3—C3—C4—C5	−128.2 (12)
O1—Cd1—O3—C2	−15.6 (4)	C4'—C3—C4—C5	−65 (6)
O3^i^—Cd1—O3—C2	−115.7 (5)	O3—C3—C4—C9	49.7 (12)
O2^ii^—Cd1—O3—C3	−56.3 (6)	C4'—C3—C4—C9	113 (7)
O2^iii^—Cd1—O3—C3	−174.5 (5)	C3—C4—C5—C6	178.0 (15)
O1^i^—Cd1—O3—C3	31.7 (5)	C3—C4—C9—C8	−177.8 (16)
O1—Cd1—O3—C3	−160.2 (6)	O3—C3—C4'—C5'	−97.6 (15)
O3^i^—Cd1—O3—C3	99.7 (5)	C4—C3—C4'—C5'	141 (8)
Cd1—O1—C1—O2	166.5 (5)	O3—C3—C4'—C9'	80.5 (10)
Cd1—O1—C1—C2	−12.5 (9)	C4—C3—C4'—C9'	−41 (7)
Cd1^iv^—O2—C1—O1	−25.2 (10)	C3—C4'—C5'—C6'	178.1 (17)
Cd1^iv^—O2—C1—C2	153.9 (4)	C3—C4'—C9'—C8'	−178.1 (17)
C3—O3—C2—C1	163.3 (6)		

Symmetry codes: (i) −*x*+2, −*y*+2, *z*; (ii) *x*−1/2, −*y*+3/2, −*z*; (iii) −*x*+5/2, *y*+1/2, −*z*; (iv) *x*+1/2, −*y*+3/2, −*z*.
